# The Relationship between All-Cause Dementia and Acute Diabetes Complications among American Indian and Alaska Native Peoples

**DOI:** 10.3390/ijerph21040496

**Published:** 2024-04-18

**Authors:** Xiaoyi Niu, Jenny Chang, Maria M. Corrada, Ann Bullock, Blythe Winchester, Spero M. Manson, Joan O’Connell, Luohua Jiang

**Affiliations:** 1 Department of Epidemiology & Biostatistics, University of California Irvine, Irvine, CA 92697, USA; xiaoyiniu58@gmail.com (X.N.); mcorrada@uci.edu (M.M.C.); 2 Department of Medicine, University of California Irvine, Irvine, CA 92697, USA; jjchang@hs.uci.edu; 3 Department of Neurology, School of Medicine, University of California Irvine, Irvine, CA 92697, USA; 4 Formerly with the Division of Diabetes Treatment and Prevention, Indian Health Service, Rockville, MD 20857, USA; akbullock2@gmail.com; 5 Cherokee Indian Hospital, Cherokee, NC 28719, USA; blythe.winchester@cherokeehospital.org; 6 Centers for American Indian and Alaska Native Health, Colorado School of Public Health, University of Colorado Anschutz Medical Campus, Aurora, CO 80045, USA; spero.manson@cuanschutz.edu (S.M.M.); joan.oconnell@cuanschutz.edu (J.O.)

**Keywords:** Alzheimer’s disease, American Indian or Alaska Native, dementia, diabetic ketoacidosis, hypoglycemia, hyperglycemia

## Abstract

Background: American Indian and Alaska Native people (AI/AN) bear a disproportionate burden of diabetes. Growing evidence shows significant associations between several acute diabetes complications and dementia among diabetes patients. However, little is known about these relationships among AI/AN adults. Here, we aim to investigate these associations among AI/AN adults. Methods: This cross-sectional study extracted data from the Indian Health Service’s (IHS) National Data Warehouse and related administrative databases. A total of 29,337 IHS actual users with diabetes who were 45+ years old during fiscal year 2013 were included. All-cause dementia and diabetes complications were identified using ICD-9 diagnostic codes. Negative binomial regression models were used to evaluate the associations of interest. Results: Nearly 3% of AI/AN diabetes patients had a dementia diagnosis. After controlling for covariates, dementia was associated with a 94% higher rate of severe *hypoglycemia* (Incidence Rate Ratio [IRR = 1.94, 95% CI:1.50–2.51), 52% higher rate of severe *hyperglycemia* (IRR = 1.52, 95% CI, 1.11–2.08), and 92% higher rate of any acute complication (IRR = 1.92, 95% CI:1.53–2.41). Conclusions: AI/AN diabetes patients with dementia suffered from considerably higher rates of acute diabetes complications than their counterparts without dementia. The clinical management of patients with comorbid diabetes and dementia is particularly challenging and may require individualized treatment approaches.

## 1. Introduction

As the US population ages, the number of people living with dementia is projected to at least double by 2050. Although the life expectancy of American Indian and Alaska Native peoples (AI/AN) is shorter than that of other US populations, it had increased dramatically in the last few decades before 2020 [1]. Thus, the prevalence of dementia is also projected to increase dramatically in this population in the next few decades. However, our knowledge about dementia in this population has failed to keep pace. Indeed, only a handful of studies have examined dementia among AI/AN adults. Recent Kaiser Permanente Northern California studies documented major racial/ethnic inequalities in both dementia incidence and survival over 14 years in their large, integrated health care delivery system [2,3,4]. Among their members aged 64+ years, AI/AN patients had substantially higher dementia incidence and shorter survival time after diagnosis than most other racial/ethnic groups [2,3]. Moreover, among those Kaiser patients with type 2 diabetes (T2D), age-adjusted dementia incidence was highest among AI/AN patients, with a 64% greater risk of developing dementia in 10 years than Asians, the racial group with the lowest dementia risk [4].

Compared to other US populations, AI/AN populations bear a disproportionate burden of many risk factors for dementia, such as diabetes, obesity, and tobacco use [5,6]. Notably, AI/AN adults have the highest rates of diabetes in the nation: adult prevalence was 14.7% in 2017, nearly twice that of the White population [7]. Substantial evidence has shown that persons with diabetes have an increased risk of dementia [8,9,10]. Growing evidence indicates that dementia is associated with several acute diabetic complications, including severe *hypoglycemia* and *hyperglycemia* [11,12,13,14,15,16,17,18,19,20]. Previous studies support bidirectional relationships between *hypoglycemia* and dementia, revealing that episodes of severe *hypoglycemia* are associated with an increased future risk of future cognitive impairment and dementia [11,14,15,16,19]; meanwhile, patients with dementia or poor cognitive function experience a higher risk of severe *hypoglycemia* [13,17,18]. Similarly, emerging evidence suggests severe *hyperglycemia* [12] and diabetic ketoacidosis (DKA) [21,22], a potentially fatal complication characterized by *hyperglycemia*, hyperketonemia, and metabolic acidosis, may be a potential risk factor for dementia. However, very few studies have investigated if patients with dementia are at higher risk for severe *hyperglycemia* or DKA. Furthermore, none of the previous studies considered these associations among AI/AN adults, a population that bears a particularly high burden of diabetes.

Many AI/AN peoples access health care through the Indian Health Service (IHS) system, which is severely under-resourced and challenged by the high morbidity burden and low socioeconomic status of its patient population. Based on treaties and subsequent legislation, the US government has a trust responsibility to provide health care to members of federally recognized Tribes, an obligation fulfilled by the IHS since 1955 [23]. The IHS includes hospitals, clinics, and health programs operated by IHS, Tribal organizations through contracts and compacts with IHS, and urban Indian clinics funded by the IHS. In 2015, the IHS reported per capita health spending was USD 3688 [24], dramatically lower than that for the US general population in 2015 (USD 9994) [25]. Further, AI/AN adults using IHS services, as compared to other racial/ethnic groups, are more likely to live in rural areas and be uninsured. Thus, while AI/AN experience some of the greatest health disparities in the US, fewer resources are allocated to prevent and treat these conditions in the meantime. Focusing on a national sample of Indian Health Service (IHS) service users, this study explored the relationship between dementia and three acute diabetic complications in AI/AN adults: *hyperglycemia*, *hypoglycemia*, and DKA. We hypothesized that diabetes patients with dementia would have increased rates of all three acute diabetic complications (the term “complications” will be utilized hereafter to refer to these acute conditions).

## 2. Methods

### 2.1. Data Source

Roughly 30% of individuals who identify as AI/AN in the US census [26] access health care via facilities funded by the IHS, encompassing hospitals, clinics, and health programs operated by the IHS (I), Tribal organizations (T), and urban Indian health programs (U). Collectively referred to as I/T/Us, these facilities provide health care to approximately 2.6 million AI/AN [27]. Primarily focusing on primary and preventive care, I/T/U facilities provide direct services whenever feasible. In cases where services are unavailable, AI/AN may be directed to alternative providers through the Purchased/Referred Care (PRC) program.

Our research drew data from the IHS Improving Health Care Delivery Data Project (IHS Data Project), a comprehensive data infrastructure housing health status, service utilization, and treatment cost information for over 640,000 AI/AN, representing approximately 30% of AI/AN utilizing IHS services [28]. Launched in 2010, the I/T/U Data Project aims to equip the IHS, Tribal leaders, and AI/AN communities with insights into the health requirements of AI/AN populations to facilitate the identification and prioritization of health promotion and resource allocation strategies. The project encompasses a purposive sample of AI/AN residing in 15 IHS Service Units (project sites), located across the country. These include one site in the East, four in the Northern Plains, two in the Southern Plains, five in the Southwest, two on the Pacific Coast, and one in Alaska [29]. During the study period, there was minimal utilization data for the urban Indian clinics; hence, in our study, we designate the providers as IHS and Tribal (I/T, instead of I/T/U) providers. Specifically, this data infrastructure combines existing electronic health record data from multiple IHS platforms spanning seven years (FY2007–2013). The data for our study encompass registration, demographic, health coverage, and I/T service utilization data retrieved from the National Data Warehouse (NDW), along with service utilization data paid for by I/T, but provided by non-I/T facilities through the PRC program. Further elaboration on this data infrastructure is available elsewhere [28].

Project personnel partner with the IHS and the Tribal organizations that participate in the IHS Data Project. This collaboration takes place through the project’s Collaborative Network, which includes meetings of three advisory committees (i.e., Steering, Project Site, and Patient), travel to the project sites, and a process to obtain approvals from the IHS National Institutional Review Board (IRB), Tribal IRBs, and Tribal Authorities in addition to the Colorado Multiple IRB.

### 2.2. Study Population

The IHS Data Project population is comparable to the national IHS service population in terms of age and sex. The study population of the current analysis included AI/AN adults with diabetes who were 45+ years old and used I/T services in FY2013. Among these adults, diabetes was identified from service utilization data in the IHS Data Project from FY2007 to 2013. We identified diabetes patients among the I/T users by one inpatient diagnosis code for diabetes, or a combination of two of the following events occurring within 24 months of each other [30]: (1) HbA1c ≥ 6.5%; (2) an outpatient diagnosis code for diabetes; or (3) any filled prescription of antihyperglycemic medication. The International Classification of Diseases Ninth Revision, Clinical Modification (ICD-9) diagnostic codes that were used to determine diabetes included 249.00–249.92, 250.x, 357.2, 366.41, and 362.01–362.08. Once identified, the diagnosis is carried forward for subsequent years. Data from 4 of the 15 sites were excluded because 2 sites did not provide I/T hospital inpatient and emergency services, 1 site had incomplete PRC hospital data, and another site was not part of the IHS data project until FY2011. A total of 29,337 diabetes patients were included in this analysis (Supplemental Figure S1).

### 2.3. Measures

Severe *hypoglycemic* and *hyperglycemic* events were defined as hospital admissions or emergency department (ED) visits with a principal discharge diagnosis code indicating *hyperglycemia* (ICD-9 codes used: 250.02, 250.03, 250.1, 250.2, 250.3) [31] or *hypoglycemia* (ICD-9 codes used: 251.0, 251.1, 251.2, 270.3, 775.0, 775.6, 962.3, 250.8, excluding visits/admissions with the secondary ICD-9 codes 259.8, 272.7, 681.xx, 682.xx, 686.9x, 707.xx, 709.3, 730.0–730.2, or 731.8) [31,32]. Cases of DKA were identified as hospitalizations or ED visits with a principal discharge diagnosis code of 250.10, 250.11, 250.12, or 250.13 [33]. Detailed definitions of each complication and the corresponding ICD-9 codes are presented in Supplemental Table S1a. For each of these 3 types of events, we calculated the total number of inpatient admissions and ED visits for *hypoglycemia*, *hyperglycemia*, or DKA from FY2007 to 2013. Adults were classified as having dementia if they possessed at least one relevant ICD-9 diagnostic code within their National Data Warehouse (NDW) or Purchased/Referred Care (PRC) inpatient and outpatient service utilization records between FY2007 and 2013. These qualifying ICD-9 codes comprised those for Alzheimer’s disease, vascular dementia, Lewy body dementia, frontotemporal dementia, alcohol-induced dementia, and other dementia types utilized in a recent Medicare study (refer to Supplemental Table S1b) [34]. Possible confounding factors that could impact the relationship between dementia and acute diabetic complications include the following: (a) demographics such as age and sex; (b) service region and health care coverage aside from IHS, including Medicaid, Medicare, and private insurance; (c) coexisting medical conditions; (d) clinical measurements (details below); and (e) usage of medications. Chronic comorbidities were identified using ICD-9 codes recorded in inpatient and outpatient service utilization records from FY2007 to 2013, supplemented by medication data. Sightlines^TM^ DxCG Risk Solutions software (Version 4.0.1, Verisk Health, Inc: Jersey City, NJ, USA) groups ICD-9 codes into Diagnostic Cost Groups (DCGs); DCGs are employed by the federal government and private insurers to identify chronic conditions [35]. We used this software to identify adults with one or more types of comorbidities: cardiovascular disease (CVD), malignant cancer, hypertension, liver disease, mental illness other than depression, depression, alcohol and drug use disorders, and tobacco use disorder. A comorbidity index was created by calculating the total number of comorbid conditions each patient had. NDW data also include several clinical measurements such as blood pressure, glycosylated hemoglobin (HbA1c), and low-density lipoprotein cholesterol (LDL) level. The Generic Product Identifier (GPI) system [36], a 14-character hierarchical classification system, was used to group NDW data on dispensed medications into types of medications by primary therapeutic use: lipid-lowering medications, antihypertensive medication, and diabetes medication (insulin only, oral only, insulin and oral combined, none). Medication data were missing from 2 project sites due to changes in their electronic health record systems.

### 2.4. Statistical Analysis

Demographic and clinical characteristics of the study sample were described by frequency distribution for categorical variables and mean and standard deviations for continuous variables. We employed two-sample *t*-tests for continuous variables and chi-square tests for categorical variables to compare patients with vs. without dementia and patients with vs. without complications. Negative binomial regression assessed the association between dementia and number of acute diabetes complications, controlling for potential confounding variables. We fitted a series of regression models to evaluate the associations of interest and understand how groups of covariates, when added sequentially to the models, influenced the estimated associations. Based on the previous literature and the availability of data in the current study, the potential confounding variables we added to the models include (a) age and sex (Model 1); (b) Model 1 plus health coverage and service region (Model 2); (c) Model 2 plus FY2013 comorbidity index (Model 3); (d) Model 3 plus FY2013 clinical measurements (Model 4); and (e) Model 4 plus FY2013 prescribed medications for selected conditions (Model 5). Both clinical measurements and prescribed medications had substantial missing data. Therefore, Model 3 is deemed as the main regression model for this study. Further, instead of excluding participants with missingness on those measures from Model 4 and 5, we coded each of the clinical measurements as categorical variables (HbA1c < 8% or ≥8% or unknown; LDL < 100 mg/dL or ≥100 mg/dL or unknown; systolic blood pressure < 140 mmHg or ≥140 mmHg or unknown) and similarly coded the medication variables into 3 categories: yes, no, or unknown. A two-sided *p* value < 0.05 was considered statistically significant. All statistical analyses were performed on SAS 9.4. (SAS, Inc., Cary, NC, USA).

## 3. Results

As shown in Table 1, of the 29,337 AI/AN diabetes patients aged 45+ years, 843 (2.87%) were identified as having a dementia diagnosis. Compared to non-dementia patients, dementia patients were older, more likely to be covered by Medicare and Medicaid, and more likely to have comorbidities. They were also more likely to be prescribed insulin as a diabetes treatment, but less likely to be prescribed oral diabetic medication and lipid-lowering medication. They had significantly lower LDL levels than those without dementia.

Table 2 compares characteristics between patients with vs. without acute diabetes complications. Over the 7 years, among AI/AN diabetes patients, similar proportions of patients had a history of hospitalization or ED visits for *hypoglycemia* only (3.6%) and *hyperglycemia* only (4.1%). About 0.8% had both types of acute diabetes complications. Among the four subgroups, patients with both *hypoglycemia* and *hyperglycemia* had the highest prevalence for most comorbidities, including CVD, liver disease, mental illness other than depression, depression, alcohol and drug use disorder, and dementia. These patients were also more likely to be prescribed insulin only. On the other hand, patients who experienced only *hyperglycemia* tended to be younger, had higher HbA1c levels, and were more likely to be prescribed a combined diabetes medication regimen. In general, patients with acute complications, regardless of type, were more likely to suffer from comorbidities and more likely to receive dyslipidemia, diabetes, and antihypertension medication prescriptions than patients without complications.

Table 3 presents the proportions of patients with acute diabetes complications by dementia status. Among dementia patients, 15.4% experienced severe *hypoglycemia* from FYs 2007–2013, which was more than three times the proportion of non-dementia patients (4.0%). The prevalence of *hyperglycemia* among dementia patients was also significantly higher than that in non-dementia patients (6.9% vs. 4.8%, *p* = 0.007). In the stratified analysis, the difference patterns of *hypoglycemia* were similar within each age and sex stratum. However, the proportion of dementia patients with severe *hyperglycemia* was significantly higher than that among non-dementia patients in females and younger age groups (<75 years old), but the proportion did not differ by dementia status in males and the oldest age group (75+ years old). The difference in the prevalence of DKA between dementia and non-dementia patients was not statistically significant. However, after stratification, we observed a significantly higher prevalence of DKA in dementia patients than non-dementia patients among females (1.7% vs. 0.7%, *p* = 0.023) and in the 65–74 age group (2.9% vs. 0.4%, *p* = 0.0012).

**Figure 1:**
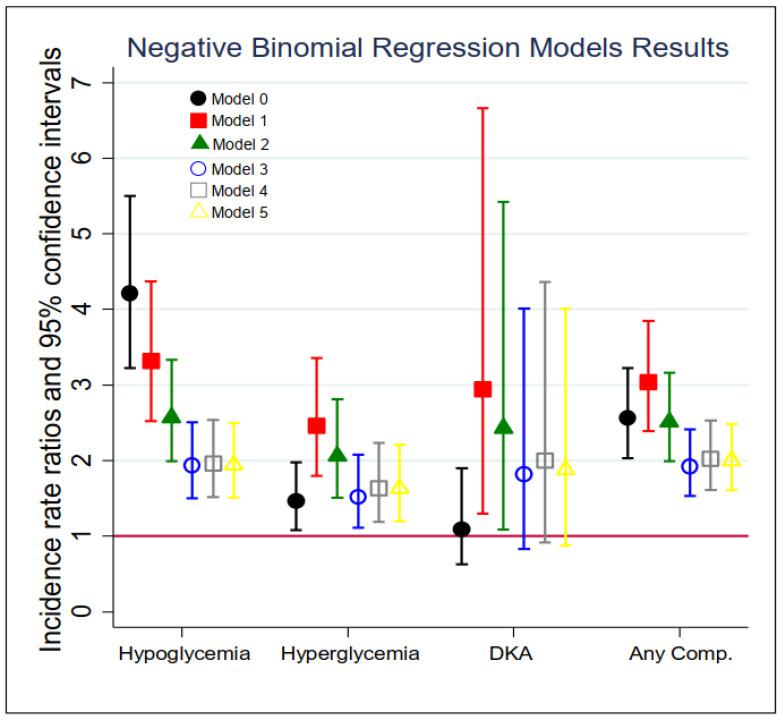
Association between of each acute diabetic complication and dementia status in fiscal year 2013 (*n* = 29,337). Models: 0 = unadjusted, dementia status only; 1 = Model 0 + demographics; 2 = Model 1 + insurance and IHS sites; 3 = Model 2 + comorbidity index (including CVD, cancer, hypertension, liver disease, mental disease, depression, alcohol and drug use disorder, and tobacco use disorder); 4 = Model 3 + clinical measurement (i.e., LDL, A1C, and SBP); 5 = Model 4 + treatment (i.e., CVD medication, diabetes medication type, hypertension medication).

The results of the negative binomial regression models for the total number of inpatient admissions and ED visits of acute diabetes complications are summarized in Figure 1 and Supplemental Table S2. In the unadjusted models (Model 0), diabetic patients with dementia were three times more likely (Incidence Rate Ratio (IRR) = 4.21, 95% CI: 3.22–5.50) to experience a *hypoglycemic* episode and about 50% more likely (IRR = 1.46, 95% CI: 1.08–1.98) to experience a *hyperglycemia* episode in the past 7 years compared to those who did not. In Model 1, after controlling for patients’ age and sex, the incidence rate of experiencing a *hypoglycemia* episode for dementia patients was 3.32 times (95% CI: 2.52–4.37) that among non-dementia patients; similarly, the risk of having a *hyperglycemia* episode for dementia patients was 2.46 times (95% CI: 1.80–3.36) that among non-dementia patients, and the risk of experiencing DKA for dementia patients was 2.94 times (95% CI: 1.30–6.66) that for non-dementia patients. In Model 3 (i.e., the main model), while adjusting for demographics, health insurance status, service region, and comorbidities, the IRR of dementia was attenuated but still significant for severe *hypoglycemia* (IRR = 1.94, 95% CI: 1.50–2.51) and *hyperglycemia* (IRR = 1.52, 95% CI: 1.11–2.08). However, the association between dementia and DKA was no longer statistically significant (IRR = 1.82, 95% CI: 0.83–4.01). Further adjustments on clinical measurements and medications did not substantially change the estimated IRRs for *hypoglycemia* and *hyperglycemia*. In the last column of Supplemental Table S2, the relationship between dementia and the total number of all types of acute diabetes complications was examined. After controlling for all the covariates (Model 5), the risk of experiencing any acute diabetic complication in the past 7 years among dementia patients was 100% higher than that among those without dementia (IRR = 2.00, 95% CI: 1.61–2.48).

## 4. Discussion

Our results show that, after adjusting for demographics, health care coverage, and comorbidities, AI/AN diabetes patients with dementia had an approximately 94% higher risk of experiencing severe hypoglycemic episodes and a 52% higher risk of experiencing severe hyperglycemic episodes than those without dementia. The positive association between dementia and *hypoglycemia* is consistent with previous studies among older adults with diabetes. A rich body of research shows an association between history of severe hypoglycemic episodes and increased risk of developing future dementia [11,14,15,16,19,37]. Meanwhile, emerging evidence indicates the relationship between dementia and hypoglycemia could be bidirectional in that dementia patients also have a higher future risk of experiencing severe *hypoglycemia* [13,17,18,38]. A possible causal mechanism for this increased risk of *hypoglycemia* is that glycemic management among diabetes patients with comorbidities, such as dementia, can be challenging. As shown in our descriptive analysis, insulin was more likely to be prescribed to patients with dementia than those without dementia, likely due to higher rates of comorbidities in people with dementia. However, cognitive dysfunction issues might affect eating patterns and/or cause a delay in recognizing symptoms of mild *hypoglycemia* associated with insulin use, which in turn can lead to severe *hypoglycemia* [39,40].

Regarding *hyperglycemia*, although a few studies found a positive association between baseline hyperglycemic episodes and future risk of dementia [12], to our knowledge, no study has investigated the reverse direction of how dementia status affects the occurrence of severe hyperglycemic events. Furthermore, only a few studies examined the relationship between dementia and DKA, a severe and potentially life-threatening type of *hyperglycemia*. These studies reported a significant association between DKA and cognitive dysfunction among type 1 diabetes (T1D) patients [22] and an increased risk of dementia among T2D patients with a history of DKA [21]. Our results confirmed a significant cross-sectional association between dementia and severe *hyperglycemia*, as well as DKA, among AI/AN diabetes patients before adjusting for comorbidities. One possible explanation for this finding is that patients with cognitive impairment may have reduced self-management abilities and low adherence to prescribed medications [41,42], which may result in *hyperglycemia*.

The significant associations between dementia and acute diabetes complications that emerged in the current study suggest that further attention needs to be paid to glycemic management among AI/AN diabetes patients with comorbid dementia. Unfortunately, no systematic guidance currently exists for the management of diabetes among dementia patients. According to the American Diabetes Association (ADA) 2021 Standards of Medical Care in Diabetes, glycemic targets should be individualized based on key patient characteristics that affect the risks and benefits of therapy for each patient [43]. Furthermore, “Older adults with multiple coexisting chronic illnesses, cognitive impairment, or functional dependence should have less-stringent glycemic goals” [44]. These issues make it difficult to develop specific recommendations regarding glycemic goals or the frequency of glycemic monitoring for patients with both diabetes and varying degrees of cognitive impairment. Some general suggestions include the following: (1) Conduct cognitive screening in older patients with diabetes. Screening helps patients and caregivers monitor cognitive function and pay attention to diabetes self-care. It will also help clinicians adjust treatment plans based on patients’ screening results. (2) Educate diabetes patients with comorbid cognitive impairment and their caregivers about appropriate diabetes self-care, especially with respect to glycemic control, home glucose testing, and medication adherence. (3) Educate providers regarding how to develop individualized treatment plans for diabetes patients with dementia. As these patients tend to have higher risks of developing both severe *hypoglycemia* and *hyperglycemia*, a patient-centered, multi-disciplinary treatment approach is recommended to assist patients and caregivers with diabetes management. This approach should take advantage of multiple local resources where available, including dieticians, clinical pharmacists, behavioral health consultants, nurses, etc., to emphasize each patient’s individual glycemic targets and individualized care plan. It is important to note that the average A1c level of patients who experienced hypoglycemia in this study was 8.2%. Given that providers may focus primarily on HbA1c value to determine whether to intensify therapy, these results underscore the importance of inquiring about hypoglycemic symptoms and looking at patients’ blood sugar records in addition to HbA1c to appropriately individualize therapy.

This study has several strengths. First, we focused on a large well-characterized cohort of AI/AN diabetes patients from geographically diverse sites across the United States. To our knowledge, this is the first study to investigate the associations between dementia and severe *hyperglycemia*/*hypoglycemia* in a population that suffers a high burden of diabetes and has also been understudied with respect to dementia. Another strength of this study is the availability of multiple linked data sources with information on health status, service utilization, medication use, and laboratory values, which yield a wide range of potential confounders, including diagnosed comorbidity conditions, diabetes medications, and HbA1c levels.

Yet, a number of limitations warrant consideration when interpreting our results. A major limitation is the cross-sectional study design. It precludes us from identifying the temporality between dementia diagnosis and the occurrence of complications, which in turn prevents any causal conclusions. As both severe *hyperglycemia* and *hypoglycemia* are relatively rare events, longitudinal data with more years of follow-up are needed for this purpose. Furthermore, identifying dementia patients via clinical diagnostic codes likely underestimates the prevalence of dementia (both diagnosed and undiagnosed) in the population under consideration. This is particularly true for a service delivery system with resource constraints that serves AI/AN who reside in rural areas with limited access to specialists [45]. Many primary care providers do not feel fully trained to diagnose dementia or are unsure which cognitive assessments are best to use with AI/AN patients [46]. Instead, they may classify patients as having some type of mild cognitive disorder and/or refer them to a specialist. However, the patients who are referred may not see a specialist due to transportation or financial restrictions. Furthermore, if the diagnosis of dementia was made at a non-I/T facility, due to the lack of specialists within local I/T facilities, and the PRC program did not pay for the provided service, an ICD-9 code indicating dementia may not be included in these data. An underestimation of dementia prevalence not only reduces the statistical power for evaluating the relationships of interest in the current study, but may also bias the estimated association toward the null because some dementia patients may have been treated as patients without dementia in our analysis.

Other limitations of our study are similar to other studies using diagnostic codes in service utilization records. First, misdiagnosis could happen and affect our study outcomes. For example, symptoms from *hypoglycemia* and dementia share some similarities, such as agitation, increased confusion, and behavioral changes, which may cause condition misclassification [39]. Yet, it is unlikely for acute hypoglycemia causing ED visits or hospitalizations to be misclassified as dementia. Second, we cannot clearly distinguish between T1D and T2D. However, T1D is very uncommon among AI/AN. According to an algorithm we developed using ICD-9 and diabetes medications to classify T1D vs. T2D patients, <2% of the study population were T1D patients. Excluding these T1D patients from the total analytical sample had minimal impact on the parameter estimates of our final models. Third, we do not have data to determine the severity of dementia or the living settings of dementia patients to assess how closely dementia patients were monitored to detect severe *hypoglycemia* or *hyperglycemia*. Further, data were not available for some sociodemographic variables (e.g., education, income, marital status) and therefore could not be adjusted for in our regression models. Yet, given the major potential confounders already included in our models, adjusting additional covariates likely would not have substantially changed our parameter estimates. In addition, some covariates included in our regression models, such as HbA1c and LDL, had a substantial rate of missing data. Although by treating missing data as an unknown category the analytical sample sizes of Models 4 and 5 were not reduced, it precluded us from treating clinical measurements as continuous covariates in those models. However, sensitivity analyses that excluded participants with missing clinical measurements and treated those measures as continuous variables resulted in similar parameter estimates and consistent conclusions as those based on Model 3. Therefore, the associations found in this study are fairly robust. Lastly, although our results are based on data from AI/AN adults who lived in 11 project sites, representing a geographically diverse population of AI/AN, our findings may not be generalizable to AI/AN peoples who live elsewhere or who do not obtain health services through the IHS or Tribal programs. Furthermore, the patients included in this study were all AI/AN, which may limit the generalizability of our findings to other populations. However, our study included a large sample size of AI/AN, which are severely under-studied in the field of dementia research, making it a unique contribution to the literature, and may provide critical insight into the association between dementia and diabetes complications in other similarly underserved populations.

## 5. Conclusions

In summary, our study found that AI/AN diabetes patients with dementia suffered from considerably higher rates of severe *hypoglycemia* and *hyperglycemia* that led to hospitalizations and/or ED visits than those without dementia. These findings in AI/AN are consistent with those of other populations, suggesting that glucose management in patients with both conditions is particularly challenging. Our findings in this understudied population further support and underscore the importance of individualizing diabetes treatment plans, especially in patients with comorbid dementia.

## Figures and Tables

**Table 1:** Characteristics of diabetes patients by dementia status in fiscal year 2013 ^a,b^.

	All	Without Dementia	With Dementia
Total *n*	29,337	28,494	843
Age (years)	61.7 ± 10.5	61.2 ± 10.2	76.6 ± 11.1
Age categories (%)			
45–64	65.4	66.9	16.5
65–74	22.1	22.2	20.5
75+	12.5	11.0	63.0
Female (%)	56.1	56.1	57.3
Health coverage (%)			
Medicare	42.6	41.3	85.3
Medicaid	12.3	12.0	22.1
Private	14.3	14.5	7.0
Comorbidities (%)			
CVD	38.7	37.8	70.2
Cancer	6.1	6.0	10.3
Liver disease	10.7	10.6	15.1
Hypertension	86.7	86.5	94.0
Depression	24.9	24.6	36.9
Mental illness other than depression ^c^	23.9	23.4	41.8
Alcohols and drug use disorder	11.8	11.6	17.3
Tobacco use disorder	16.5	16.6	10.8
Number of comorbidities	2.2 ± 1.3	2.2 ± 1.3	3.0 ± 1.3
Medications (*n* = 24,964) (%) ^d^			
Dyslipidemia medication ^e^	55.4	55.6	48.1
Diabetes medication			
Insulin only	10.0	9.8	19.7
Oral only	40.2	40.6	26.4
Insulin and oral combined	19.8	19.9	16.4
No diabetes medication	30.0	29.8	37.5
Hypertension medication ^f^	74.8	74.8	74.5
Clinical measurements			
LDL (mg/dL) (*n* = 17,231) ^g^	92.5 ± 32.4	92.7 ± 32.4	83.8 ± 31.3
A1c (%) (*n* = 19,891)	7.9 ± 2.0	7.9 ± 2.0	7.4 ± 1.7
Systolic blood pressure (mmHg) (*n* = 22,730)	131.9 ± 15.0	131.9 ± 14.9	129.8 ± 16.8

^a^ All differences are statistically significant between dementia patients and non-dementia patients except sex and hypertension medication. ^b^ Sum of percentages might not be 100% due to rounding. ^c^ Other mental illnesses include eating disorder, mood and anxiety disorder, personality disorder, psychoses, and prolonged PTSD. ^d^ When calculating medication percentage, patients from two sites (*n* = 4373) were excluded due to incomplete medication data. ^e^ Dyslipidemia medication includes statins and other lipid-lowering medications. ^f^ Hypertension medication includes diuretics, beta blockers, calcium channel blockers, ACE inhibitors, ARBs, and other antihypertensive medications. ^g^ Because the number of patients with LDL levels between 30 and 40 mg/dL was unusually high from one site (*n* = 518), LDL levels from these patients were not used.

**Table 2:** Characteristics of diabetes patients by acute diabetes complication status in fiscal year 2013 ^a,b^.

	All	Without Any Complication	Only Hypoglycemia	Only Hyperglycemia	Both Complications
Total *n*	29,337	26,859	1043	1199	236
Age (years)	61.7 ± 10.5	61.6 ± 10.5	65.7 ± 11.0	58.4 ± 9.3	62.1 ± 11.6
Age categories (%)					
45–64	65.4	65.5	49.4	77.8	65.7
65–74	22.1	22.2	27.8	15.8	16.5
75+	12.5	12.3	22.8	6.4	17.8
Female (%)	56.1	56.1	58.3	54.1	56.4
Health coverage (%)					
Medicare	42.6	42.0	62.0	36.2	52.5
Medicaid	12.3	11.8	19.1	17.2	18.6
Private	14.3	14.6	10.3	12.0	11.0
Comorbidities (%)					
CVD	38.7	36.8	68.6	50.0	68.6
Cancer	6.1	6.0	8.4	5.9	6.4
Liver disease	10.7	10.1	16.4	17.0	18.6
Hypertension	86.7	86.2	94.6	89.7	92.8
Depression	24.9	24.3	29.9	31.7	40.7
Mental illness other than depression ^c^	23.9	23.3	30.6	29.4	36.4
Alcohols and drug use disorder	11.8	10.9	19.2	22.8	30.1
Tobacco use disorder	16.5	16.2	14.7	22.3	21.2
Dementia	2.9	2.5	9.9	2.6	11.4
Number of comorbidities	2.2 ± 1.3	2.1 ± 1.3	2.8 ± 1.3	2.7 ± 1.4	3.1 ± 1.4
Medications (*n* = 24,964) (%) ^d^					
Dyslipidemia medication ^e^	55.4	54.8	63.2	61.9	58.3
Diabetes medication					
Insulin only	10.0	8.4	24.4	25.3	41.7
Oral only	40.2	42.0	27.5	18.8	8.8
Insulin and oral combined	19.8	18.2	30.7	41.9	36.1
No diabetes medication	30.0	31.4	17.5	14.1	13.4
Hypertension medication ^f^	74.8	73.9	85.7	82.2	82.4
Clinical measurements					
LDL (mg/dL) (*n* = 17,231) ^g^	92.5 ± 32.4	92.8 ± 32.3	84.9 ± 30.8	94.6 ± 35.2	87.6 ± 36.3
A1c (%) (*n* = 19,891)	7.9 ± 2.0	7.8 ± 1.9	8.2 ± 2.1	9.4 ± 2.3	9.0 ± 2.0
Systolic blood pressure (mmHg) (*n* = 22,730)	131.9 ± 15.0	131.9 ± 14.9	131.6 ± 16.6	131.9 ± 15.6	131.7 ± 16.3

^a^ All differences are statistically significant among diabetes complication subgroups except sex and SBP. ^b^ Sum of percentages might not be 100% due to rounding. ^c^ Other mental illnesses include eating disorder, mood and anxiety disorder, personality disorder, psychoses, and prolonged PTSD. ^d^ When calculating medication percentage, patients from two sites (*n* = 4373) were excluded due to incomplete medication data. ^e^ Dyslipidemia medication includes statins and other lipid-lowering agents. ^f^ Hypertension medication includes diuretics, beta blockers, calcium channel blockers, ACE inhibitors, ARBs, and other antihypertensive medications. ^g^ Because the number of patients with LDL levels between 30 and 40 mg/dL was unusually high from one site (*n* = 518), LDL levels from these patients were not used.

**Table 3:** Proportions of diabetes patients with acute complications by dementia status in fiscal year 2013.

Diabetic Complications	Total	Without Dementia	With Dementia	*p*-Value
Overall	*n* = 29,337	*n* = 28,494	*n* = 843	
Hypoglycemia	1279 (4.4%)	1149 (4.0%)	130 (15.4%)	<0.0001
Hyperglycemia	1435 (4.9%)	1377 (4.8%)	58 (6.9%)	0.0066
Diabetic Ketoacidosis	268 (0.9%)	257 (0.9%)	11 (1.3%)	0.226
Female	*n* = 16,454	*n* = 15,971	*n* = 483	
Hypoglycemia	741 (4.5%)	662 (4.2%)	79 (16.4%)	<0.0001
Hyperglycemia	782 (4.8%)	745 (4.7%)	37 (7.7%)	0.0023
Diabetic Ketoacidosis	118 (0.7%)	110 (0.7%)	8 (1.7%)	0.0230
Male	*n* = 12,883	*n* = 12,523	*n* = 360	
Hypoglycemia	538 (4.2%)	487 (3.9%)	51 (14.2%)	<0.0001
Hyperglycemia	653 (5.1%)	632 (5.1%)	21 (5.8%)	0.502
Diabetic Ketoacidosis	150 (1.2%)	147 (1.2%)	3 (0.8%)	0.802
45–64	*n* = 19,196	*n* = 19,057	*n* = 139	
Hypoglycemia	670 (3.5%)	650 (3.4%)	20 (14.4%)	<0.0001
Hyperglycemia	1088 (5.7%)	1071 (5.6%)	17 (12.2%)	0.0008
Diabetic Ketoacidosis	222 (1.2%)	219 (1.2%)	3 (2.2%)	0.218
65–74	*n* = 6486	*n* = 6313	*n* = 173	
Hypoglycemia	329 (5.1%)	300 (4.8%)	29 (16.8%)	<0.0001
Hyperglycemia	228 (3.5%)	210 (3.3%)	18 (10.4%)	<0.0001
Diabetic Ketoacidosis	31 (0.5%)	26 (0.4%)	5 (2.9%)	0.0012
75 or more	*n* = 3655	*n* = 3124	*n* = 531	
Hypoglycemia	280 (7.7%)	199 (6.4%)	81 (15.3%)	<0.0001
Hyperglycemia	119 (3.3%)	96 (3.1%)	23 (4.3%)	0.131
Diabetic Ketoacidosis	15 (0.4%)	12 (0.4%)	3 (0.6%)	0.470

## Data Availability

The data from the IHS Data Project used to support the findings of this study have not been made available because of IHS and Tribal regulations regarding data confidentiality and security.

## Supplementary Materials

The following supporting information can be downloaded at: https://www.mdpi.com/article/10.3390/ijerph21040496/s1, Supplemental Figure S1: Flowchart of inclusion and exclusion of the study population; Supplemental Table S1a: ICD-9 Diagnoses Codes used to identify three acute diabetic complications; Supplemental Table S1b: ICD-9 Diagnoses Codes used to identify dementia in the Medicare study; Supplemental Table S2. Association between # of each acute diabetic complication and dementia status in fiscal year 2013 (*n* = 29,337).
