# Supplementary material for: The Relationship between All-Cause Dementia and Acute Diabetes Complications among American Indian and Alaska Native Peoples
Source: Int J Environ Res Public Health. 2024 Apr 18;21(4):496. doi: 10.3390/ijerph21040496 (PMC11049920; doi:10.3390/ijerph21040496)
Supplement: Supplementary file 1 [file ijerph-21-00496-s001.zip › ijerph-2886187-supplementary.pdf]

Supplemental Table S1a. ICD-9 Diagnoses Codes used to identify three acute diabetic complications

Three acute diabetic complications	ICD-9 Diagnosis Code
Hypoglycemia	251.0, 251.1, 251.2, 270.3, 775.0, 775.6, 962.3, 250.8 with excluding admissions with the secondary ICD-9 codes: 259.8, 272.7, 681.xx, 682.xx, 686.9x, 707.xx, 709.3, 730.0-730.2, or 731.8
Hyperglycemia	250.02, 250.03, 250.1, 250.2, 250.3
Ketoacidosis	250.10, 250.11, 250.12, or 250.13

Supplemental Table S1b. ICD-9 Diagnoses Codes used to identify dementia in the Medicare study

Dementia subtype	ICD-9 diagnosis code
Alzheimer's disease	331.0
Vascular	290.40, 290.41, 290.42, 290.43
Lewy Body*	331.82, 332.0 + 331.0*
Frontotemporal	331.1, 331.11, 331.19
Alcohol induced	291.2
Other†	046.11, 046.19, 292.82, 333.4
Not otherwise specified	290.0, 290.10, 290.11, 290.12, 290.13, 290.20, 290.21, 290.3, 290.9, 294.1, 294.10, 294.11, 294.20, 294.21, 294.8, 331.2, 797

* Diagnosis code 332.0 had to have a diagnosis code of 331.0 on the same claim to be considered Lewy Body.
† Other dementia includes Creutzfeldt-Jakob disease, Huntington's chorea, and drug-induced dementia.
Source: Goodman RA, Lochner KA, Thambisetty M, Wingo TS, Posner SF, Ling SM. Prevalence of dementia subtypes in United States Medicare fee-for-service beneficiaries, 2011–2013. Alzheimer's Dement. 2017; 13(1):28-37.

Supplemental Table S2. Association between # of each acute diabetic complication and dementia status in fiscal year 2013 (n=29,337)

	**Hypoglycemia (total # of events=1,901)**		**Hyperglycemia (total # of events=1,989)**		**Diabetic Ketoacidosis (total # of events=377)**		**Any Complication (total # of events=4,276)**	
	IRR (95% CI)	P-value	IRR (95% CI)	P-value	IRR (95% CI)	P-value	IRR (95% CI)	P-value
**Model 0**	4.21 (3.22, 5.50)	<.0001	1.46 (1.08, 1.98)	0.0139	1.09 (0.63, 1.90)	0.7581	2.56 (2.03, 3.22)	<.0001
**Model 1**	3.32 (2.52, 4.37)	<.0001	2.46 (1.80, 3.36)	<.0001	2.94 (1.30, 6.66)	0.0098	3.04 (2.39, 3.85)	<.0001
**Model 2**	2.57 (1.99, 3.33)	<.0001	2.06 (1.51, 2.81)	<.0001	2.43 (1.09, 5.42)	0.0300	2.51 (1.99, 3.16)	<.0001
**Model 3**	1.94 (1.50, 2.51)	<.0001	1.52 (1.11, 2.08)	0.0094	1.82 (0.83, 4.01)	0.1345	1.92 (1.53, 2.41)	<.0001
**Model 4**	1.96 (1.52, 2.54)	<.0001	1.63 (1.19, 2.23)	0.0023	2.00 (0.92, 4.36)	0.0799	2.02 (1.61, 2.53)	<.0001
**Model 5**	1.94 (1.51, 2.50)	<.0001	1.63 (1.20, 2.21)	0.0018	1.88 (0.88, 4.01)	0.1013	2.00 (1.61, 2.48)	<.0001

Models: 0=Unadjusted, dementia status only; 1=Model 0 + Demographics; 2=Model 1 + Insurance and IHS sites; 3= Model 2 + Comorbidity index (including CVD, Cancer, Hypertension, Liver disease, Mental disease, Depression, Alcohol and drug use disorder, and Tobacco use disorder); 4= Model 3 + Clinical measurement (i.e., LDL, A1C, and SBP); 5= Model 4 + Treatment (i.e., CVD medication, Diabetes medication type, Hypertension medication)
IRR, Incidence Rate Ratio (i.e., Relative Risk).

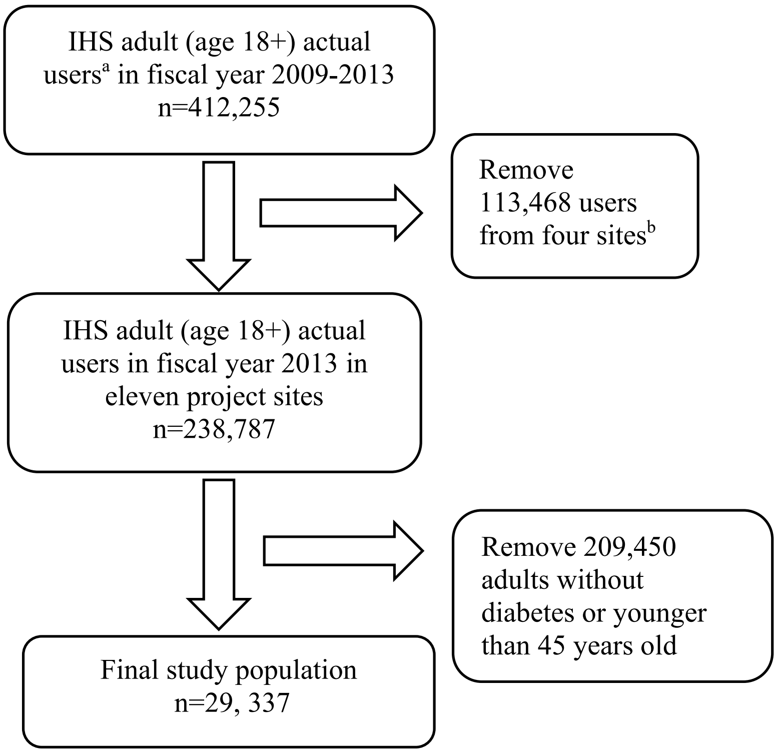

Supplemental Figure S1. Flowchart of inclusion and exclusion of the study population.

[a] Indian Health Service (IHS) actual users in a fiscal year (FY) refer to those who used IHS services at least once with a diagnostic code in that FY.

[b] Data from 4 of the 15 project sites were excluded because 2 sites did not provide I/T hospital inpatient and emergency services, 1 site had incomplete purchased/referred care (PRC) hospital data, and another site was not part of the IHS data project until FY2011.
